# Acute Effects of Adding Self-Control Tasks to the Daily Mile on Subsequent Cognition and Enjoyment in Children

**DOI:** 10.3390/healthcare14070939

**Published:** 2026-04-03

**Authors:** Anna Dunn, Grace W. M. Walters, Ryan A. Williams, Karah J. Dring, Robert Needham, Simon B. Cooper, Ruth Boat

**Affiliations:** Sport Science Department, Sport, Health and Performance Enhancement (SHAPE) Research Centre, School of Science and Technology, Nottingham Trent University, Clifton Campus, Nottingham NG11 8NS, UK; anna.dunn2017@my.ntu.ac.uk (A.D.); grace.walters@ntu.ac.uk (G.W.M.W.); ryan.williams@ntu.ac.uk (R.A.W.); karah.dring@ntu.ac.uk (K.J.D.); robert.needham@ntu.ac.uk (R.N.); simon.cooper@ntu.ac.uk (S.B.C.)

**Keywords:** self-control, the daily mile, cognition, enjoyment

## Abstract

**Highlights:**

**What is the main finding?**

- Addition of self-control tasks into The Daily Mile elicits mixed cognitive and qualitative outcomes.

**What is the implication of the main finding?**

- Tailored physical activity may optimize engagement and cognitive benefits.

**Abstract:**

**Background**: Physical activity requiring self-control may yield greater post-activity cognitive improvements. Implementing such tasks within The Daily Mile could enhance cognition further while adding variety to the initiative. This study examined whether the inclusion of self-control tasks within The Daily Mile influences subsequent cognition and enjoyment. **Methods**: Participants, numbering 99 (10.2 ± 1.1 y), completed three trials (Daily Mile Normal, Daily Mile Self-Control, and resting), using a within-subject, order-balanced, crossover design. The Daily Mile Self-Control involved students completing tasks requiring self-control for 30 s every 2 min within The Daily Mile. Cognitive tests (Stroop test, Sternberg Paradigm, Visual Search test) were administered prior to, immediately following and 45 min following The Daily Mile and resting trials. During the trials, distance covered (m), average heart rate (beats·min^−1^) and physical activity enjoyment (PACES) were measured. Focus groups explored factors affecting enjoyment during The Daily Mile trials. The effects of physical activity vs. rest on cognitive function were examined first, followed by the effect of adding self-control tasks to The Daily Mile. **Results**: There were no statistically significant differences between the Daily Mile trials on distance covered or physical activity enjoyment. However, average heart rate was significantly higher in The Daily Mile Self-Control compared to The Daily Mile Normal. Compared to rest, The Daily Mile had a positive effect on inhibitory control. Working-memory accuracy maintained following activity, but perceptual accuracy was briefly impaired. Following the addition of self-control tasks, working memory response times improved. However, accuracy on inhibitory control and perception declined immediately after activity, compared to The Daily Mile Normal. Thematic analysis indicated varied perceptions among participants, with some valuing the simplicity and control of The Daily Mile Normal, and others favoring The Daily Mile Self-Control due to the variety and cognitive challenge. **Conclusions**: Incorporating self-control tasks into The Daily Mile produced mixed cognitive and qualitative responses, compared to The Daily Mile Normal. This suggests that tailoring physical activity to individual preferences may optimize engagement and cognitive outcomes.

## 1. Introduction

In England, only 49.1% of children and young people meet the Chief Medical Officers guidelines of an average of at least 60 min of moderate-to-vigorous intensity physical activity per day [1]. Primary school children aged 7–9 (school year 3–4) and 9–11 (school year 5–6) years show the lowest activity levels, with only 41% and 46% respectively, meeting the recommendations [1]. A substantial body of research highlights the health benefits of physical activity in children, including improved sleep quality, better mental health [2], improved social skills [3], enhanced cognitive function [4] and improved concentration [5]. As children spend much of their time at school, and because large groups can be reached simultaneously, schools are attractive settings for physical activity interventions [6]. Consequently, primary school physical activity initiatives have been developed to help promote health and well-being [7]. Cognitive function refers to processes in the brain that contribute to attention, memory, problem-solving, language, perception and higher-level cognitive functioning, referred to as executive functions [8]. The core executive functions of working memory, inhibitory control and cognitive flexibility are essential for maintaining attention, problem-solving and regulating behavior [8]. A meta-analysis [9] concluded that the associations between components of executive functions and academic outcomes (reading, mathematics, language) are significant throughout elementary school. This highlights the importance of optimizing executive function to enhance academic performance and concentration.

Given the important roles of cognitive function in children, there has been much interest in the factors that can be modified to influence cognitive function in this population. One attractive intervention target to enhance cognitive performance that has received considerable attention in the literature is physical activity, particularly school-based physical activity interventions due to their ability to reach all children [6]. Encouragingly, physical activity has been shown to have a small, positive effect across a range of domains of cognition [10,11]. Many school-based physical activity interventions have been developed, but one with incredibly widespread adoption and success is The Daily Mile [12,13]. The Daily Mile involves children completing 15 min of self-paced activity each day, consisting of walking and/or running laps, typically around a school playground or playing field. Following the inception of The Daily Mile in 2012, it is now implemented in more than 20,000 schools across 96 countries [13]; thus it has significant reach. The success of The Daily Mile can be attributed to its simplistic, accessible and effective nature [12].

In terms of how The Daily Mile affects cognitive performance, Booth et al. [14] found that when children (mean age = 9 y) engaged in 15 min of self-paced physical activity similar to The Daily Mile, their cognition (inhibition, visual-spatial working memory and verbal working memory) was found to be greater, compared to a control activity of sitting/standing and a high intensity exercise (bleep test). However, research in the area is conflicting, with Martins et al. [15] observing that The Daily Mile did not improve participant’s cognitive performance (inhibitory control, visual and verbal recall), when compared to high-intensity shuttle runs or rest in 29 children (mean age = 9 y). Furthermore, Hatch et al. [16] found no acute effects of The Daily Mile on subsequent cognition (inhibitory control, visual working memory and cognitive flexibility), compared to a resting trial in 104 children aged 9–11 y. Additionally, Morris et al. [17] found there to be no significant improvements in executive function or math fluency following The Daily Mile in primary school children (aged 7–10 y). Across the existing studies, findings regarding the cognitive effects following The Daily Mile have been inconsistent, likely as a result of methodological variation. For example, Booth et al. [14] employed a moderate exercise intensity, whereas Hatch et al. [16] did not standardize the exercise intensity and allowed participants to run at a self-paced nature, to mimic real-world school settings. Additionally, Martins et al. [15] reported considerably lower intensity in The Daily Mile, compared to the high-intensity shuttle runs, which could help to explain the absence of cognitive benefits. In contrast to the previously mentioned studies, Morris et al. [17] employed a long-term Daily Mile intervention, rather than looking at the acute effects. This could explain the absence of cognitive benefits, as chronic participation alone may not be sufficient for cognitive benefits. Furthermore, the cognitive measures used across the studies varied widely in terms of domain specificity and the timings of assessment. Collectively, these methodological differences could contribute to the mixed pattern of findings reported within the literature. With the existing evidence being conflicted, it places emphasis on the requirement for further exploration of how participation in The Daily Mile acutely affects cognitive performance in children.

Evidence has shown that children find The Daily Mile enjoyable due to the self-paced nature and outdoor location [16]. However, evidence also suggests that some children’s perceptions of The Daily Mile are that it is boring; this is due to the repetitive nature of the activity [16], with evidence suggesting that not all children fully enjoy or engage in The Daily Mile [18]. Instead, participants indicated that they would prefer more varied forms of physical activity, with Scannell and Murphy [18] offering activities within The Daily Mile such as basketball shooting, rope skipping and soccer kicking. This lack of enjoyment experienced by children whilst completing The Daily Mile may lead to reductions in engagement with the initiative, thus potentially minimizing its benefits for health and cognition. Importantly, research by Fairhurst and Hotham [19] highlighted that children prioritize enjoyment when participating in physical activity. Therefore, it is imperative that research identifies ways to prevent boredom, and enhance enjoyment levels of The Daily Mile, to maximize its full potential.

One form of physical activity that children have reported enjoying is activity that requires an element of cognitive engagement [18,20]. Cognitively engaging activity refers to physical activities that incorporate tasks requiring focused attention, self-control, memory and problem-solving [21,22]. More specifically, many of the cognitively engaging physical activities that have been used in previous studies, such as “Simon Says”, require self-control (defined as the ability to volitionally override dominant responses to reach long-term goals) [23]. Self-control, often referred to as inhibitory control, is a core component of executive function and a gateway to other executive functions (e.g., working memory and cognitive flexibility) [24]. Given that executive function is strongly related to academic outcomes [25], targeting this domain during the critical school age is essential. Furthermore, self-control tasks are practical and easily integrated into children’s physical activity, due to familiarity and simplicity [26]. Such physical activities requiring self-control have been shown to produce greater post-activity improvements in cognitive function compared to activity with less cognitive engagement [22,27,28]. Therefore, it is possible that incorporating self-control tasks into The Daily Mile could both optimize the cognitive benefits and introduce a more varied form of activity to the initiative, subsequently improving enjoyment and reducing boredom. No studies to date have examined whether implementing self-control within The Daily Mile enhances cognition. Primary school age children are in a key developmental window for executive functions, especially self-control [8]. Thus, engaging in physical activities that incorporate self-control may therefore align closely with the cognitive abilities that are rapidly developing during this stage of development.

Therefore, the present study examines the potential for physical activities requiring self-control, to optimize the cognitive benefits of The Daily Mile. This is carried out through a randomized crossover trial comparing The Daily Mile (Normal), The Daily Mile with self-control activities added, and a resting control trial. Furthermore, the perceptions of children will be explored to gain a better understanding of the factors that influence their motivation and enjoyment. Thus, the aims of the current study are three-fold: (1) To examine whether an acute bout of activity (in the form of The Daily Mile, with or without self-control tasks) affects subsequent cognition (inhibitory control, visual working memory and perception) compared to a resting control; (2) To determine whether the inclusion of self-control physical activity tasks within The Daily Mile influences subsequent cognition compared to activity without cognitive engagement (The Daily Mile Normal); (3) To better understand children’s perceptions of participating in The Daily Mile, and whether the inclusion of self-control physical activity tasks within The Daily Mile influence their perceptions and enjoyment. Based on these aims, the following hypotheses were formulated. It was hypothesized that an acute bout of The Daily Mile (with or without self-control tasks) would lead to improved cognition when compared to a resting control. It was further hypothesized that incorporating self-control tasks into The Daily Mile would produce greater cognitive benefits compared to The Daily Mile Normal. Finally, it was hypothesized that children would report positive perceptions of participating in The Daily Mile, particularly more so with the inclusion of self-control tasks.

## 2. Method

### 2.1. Participant Characteristics

Following favorable opinion granted from a University Ethical Advisory Committee, primary schools in the East Midlands area were invited to participate in the study. Ninety-nine participants (49 male), aged 8–11 y from six primary schools, were recruited on a voluntary basis. The sample consisted of 29 students from year 4 (age 8–9 y), 23 from year 5 (9–10 y), and 47 students from year 6 (10–11 y). None of the schools participating implemented The Daily Mile initiative prior to participating in the study. Headteacher consent was obtained in addition to written informed consent from parents/guardians. To determine eligibility to participate in the study, parents/guardians were asked to complete a health screen questionnaire, with any participants having underlying health conditions (e.g., heart condition) that may have affected participation in the study being excluded. Participant’s written assent was gained following a verbal explanation of the study at the beginning of the familiarization trial. Participants and parents/guardians were provided with the opportunity to ask questions to clarify any element of the study that they did not fully understand.

### 2.2. Study Design

Utilizing a within-subjects, order-balanced crossover design, the study consisted of 4 visits; visit 1 being the familiarization trial, and visits 2, 3, and 4 being one of the three experimental trials (The Daily Mile Normal, The Daily Mile Self-Control, and resting control trial). Upon arrival at the familiarization trial (~9.00 am), anthropometric measurements were taken, consisting of height, sitting height, body mass and waist and hip circumference (see measures). Participants were familiarized with the cognitive function test battery twice and completed the multi-stage fitness test. At the end of the familiarization session, participants were familiarized with the self-control tasks and The Daily Mile. The familiarization trial took place 7 d prior to visit 1, with the three experimental trials each separated by 7 d.

### 2.3. Pre-Trial Control

Participants were asked to fast from 9 pm the night before each of the three experimental trials and arrive at the session fasted, with water consumption allowed *ad libitum*. In addition, participants were asked to refrain from vigorous physical activity that was not part of their usual routine. A member of the research team contacted parent/guardians on the evening prior to each experimental trial, ensuring compliance to the requirements. Upon arrival at the experimental sessions (~9.00 am), participants were provided a standardized breakfast, consisting of cornflakes, milk, toast and margarine. The breakfast provided 1.5 g of carbohydrate per kg of body mass. A dietary control was implemented to check for the effect of breakfast and the interaction effects of breakfast and exercise on subsequent cognition [29,30]; this was employed in studies of a similar nature [16].

### 2.4. Physical Activity and Control Protocol

**Figure 1:**
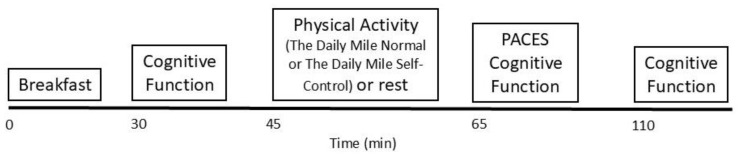
Experimental protocol.

The Daily Mile Normal trial consisted of participants completing The Daily Mile, involving 15 min of self-paced activity outdoors (school playground and school field). The Daily Mile Self-Control trial consisted of 15 min self-paced activity, with self-control tasks implemented throughout, in 30 s blocks every 2 min. Participants were encouraged to continue moving at their chosen pace throughout The Daily Mile Self-Control trial, including during the self-control tasks. In both The Daily Mile Normal and The Daily Mile Self-Control trials, the activity was designed to replicate the activity that occurs in The Daily Mile initiative. During the control trial, participants were sat in a classroom and conversed in a calm manner with their peers, ensuring that they did not engage in any activity that raised their heart rate. See Figure 1 for a schematic of the experimental protocol.

#### 2.4.1. The Daily Mile

The Daily Mile consisted of 15 min of activity at participant’s chosen pace (e.g., walk, jog or run). The physical activity was completed outside and involved completing laps of a sports pitch or playground area, in line with previous studies on The Daily Mile [16,31]. For The Daily Mile Self-Control trial, participants completed 2 min of The Daily Mile Normal followed by 30 s of a self-control game; this sequence was repeated 6 times to give an equal duration of activity (15 min) to The Daily Mile Normal trial.

#### 2.4.2. Self-Control Tasks

Three tasks requiring participants to engage their self-control were implemented in short blocks (30 s every 2 min) throughout The Daily Mile Self-Control trial. Tasks included ‘Simon Says’, whereby the researcher took the role of “Simon” and issued instructions to the participants. These were to be followed only when preceded with the phrase “Simon Says”. The second task implemented was ‘Run–Walk’. Participants were instructed to run when ‘walk’ was commanded and walk when ‘run’ was commanded. The third task was ‘Body part mix up’. Researchers informed participants of the rules prior to starting The Daily Mile activity (e.g., “head” = touch toes, “toes”= touch head). Similar tasks were adopted in previous work [32], are appropriate for the age of participants, and have been shown to require self-control [33]. Each self-control task was completed twice in the 15 min activity period. Participants were encouraged to continue moving when possible whilst completing the self-control tasks.

### 2.5. Measures

#### 2.5.1. Anthropometrics

Height was measured using a Leicester Height Measure (Seca, Hamburg, Germany), accurate to 0.1 cm and a Seca 770 digital scale (Seca, Hamburg, Germany), accurate to 0.1 kg, was used to measure body mass. These measurements were used in the determination of body mass index (BMI) and BMI percentile [34]. Sitting height measurements were taken to determine an estimation of maturity offset (by calculating years from peak height velocity) [35]. Waist circumference was measured at the narrowest point of the torso, between the xiphoid process of the sternum and the iliac crest. Hip circumference was measured at the widest part of the hips. Two measurements were taken for hip and waist circumference, with the mean as the criterion value. If there was a discrepancy by >5%, a third measure was taken and the median value used as the criterion. Identical methods were used successfully in previous research [16,30]. These data were used for participant descriptive purposes. Participant characteristics are displayed in Table 1.

#### 2.5.2. Multi-Stage Fitness Test

To assess physical fitness, the multi-stage fitness test (MSFT) [36] was completed. The test involved progressive 20 m shuttle runs, in time with an audio signal that increased by 0.14 m·s^−1^ every minute. Participants completed the test until volitional exhaustion, or until they were unable to keep in time with the audio signal. A member of the research team paced the test, and participants were provided verbal encouragement throughout from members of the research team to encourage maximum effort. Following completion of the MSFT, the participant’s score (level completed) was translated into distance covered (m) which was used as the criterion measure [37].

#### 2.5.3. Cognitive Function Tests

Participants undertook a cognitive function test battery, consisting of a Stroop test [38], Sternberg Paradigm [39], and the Visual Search test. The tests lasted approximately 15 min and were administered on a laptop computer. Prior to the start of each level, participants completed 3–6 practice stimuli to re-familiarize with the test and negated any potential learning effects. Data for these stimuli were discarded. The battery was completed by participants prior to activity, immediately following activity and 45 min following activity. Participants completed the tests in a silent classroom with lights dimmed and were provided with sound cancelling headphones to minimize distractions. Participants were instructed to complete the tasks as quickly as possible, but also as accurately as possible. The variables of interest from each test were response time (ms) of correct responses and the proportion (%) of correct responses. This test battery was utilized successfully in previous similar work [16].

The Stroop test is a measure of attention and executive function (self-control). The Stroop test consisted of two levels. On the first level (simple), a word (always a color) appeared in the center of the screen, and the participant had to choose either the word on the left or right of the screen that matches the central word, using the arrow keys. Both the target word and test word were displayed in white font. On the second level (complex), the participant had to select the color the word was written in, rather than the word itself (e.g., if ‘blue’ was written in yellow font, the correct response would be yellow), again by choosing from the words on the left and right of the screen, using the arrow keys. The simple level consisted of a total of 20 stimuli, whereas the complex level consisted of 40 stimuli. The response options remained on the screen until the participant made a selection, after which a one-second inter-stimulus interval was presented.

The Sternberg Paradigm is a measure of working memory and involves three levels: one item, three item, and five item. On each level, an item (number or letter) appeared on the screen and participants were required to select whether the presented item is one of their ‘target’ items (by pressing the right arrow key) or a distractor (by pressing the left arrow key). The one item level consisted of 16 test stimuli, with the number ‘3’ as the target. Both the three item and five item levels contained 32 test stimuli, with the target items containing either three (e.g., ‘F T Y’) or five (e.g., ‘R M D S G’) randomly generated letters.

The Visual Search Task is a measure of visual perception and involves two levels: simple and complex. Participants were instructed to respond as quickly as possible to the stimuli by pressing the spacebar on the keyboard. The simple level consists of triangles drawn in solid green lines on a black background, with 20 targets requiring responses. The complex level has random green dots covering the screen, redrawn every 250 ms to induce a flickering background effect. Triangles were drawn with a few dots and increased in density until the participant responded. The complex level contained 40 targets.

#### 2.5.4. Heart Rate

To measure physical activity intensity during participation in The Daily Mile Normal and The Daily Mile Self-Control, heart rate was recorded using a chest worn heart rate monitor (Firstbeat Technologies Ltd., Jyväskylä, Finland). Peak and average heart rate were recorded during the trials and expressed as a percentage of maximal predicted heart rate. Maximal predicted heart rate was determined using a calculation by Tanaka, Monahan and Seals [40], and validated in children [41].

#### 2.5.5. Global Positioning System

During participation in The Daily Mile Normal and The Daily Mile Self-Control trials, PlayerTek Global Position System (GPS; Catapult Sports, Melbourne, Australia) units were worn by participants. Prior to administering, the GPS units were switched on outside. The mean satellite signal strength was 9 ± 1 and the horizontal dilution of precision was 1.00 ± 0.15. Participants were fitted with an elasticated harness, which held the GPS units, placed between the scapulae. The variable of interest for the trials was total distance covered (m) during the 15 min activity period.

#### 2.5.6. Physical Activity Enjoyment

To assess participant enjoyment post-physical activity (The Daily Mile Normal and The Daily Mile Self-Control), a revised version of the Physical Activity Enjoyment Scale (PACES) was utilized [42]. The scale consists of 16 statements which originally begin with “When I am physically active…,” which are adapted to “When I am taking part in The Daily Mile sessions…,” followed by a statement (e.g., “I enjoy it”). The questionnaire was administered immediately post-physical activity. This questionnaire was previously used in a similar population [43]. Items were rated on a 5-point Likert scale (1 = “disagree a lot” to 5 = “agree a lot”). Total activity enjoyment was calculated by summing the 16 responses (seven of which were reverse scored), resulting in a range of 16–80, with higher scores reflecting higher enjoyment of physical activity.

#### 2.5.7. Focus Groups

Following completion of The Daily Mile Normal and The Daily Mile Self-Control trials, focus groups (duration: 13 ± 3 min), with 5–7 participants in each group, were utilized to explore the participant’s perceptions and enjoyment during The Daily Mile Normal and The Daily Mile Self-Control. Examples of the questions asked included ‘Would you do The Daily Mile Self-Control again, and why?’, ‘Did you prefer The Daily Mile with or without self-control tasks, and why?’ and ‘Were you more motivated to run when the self-control tasks were included?’ Focus groups took place in a quiet classroom in the school, to minimize distractions. Focus groups were selected as they have been demonstrated to be an appropriate method of gaining insight into children’s perceptions and beliefs [44], with children enjoying being asked their opinions [45]. Questions asked were open-ended and the researcher followed a semi-structured guide to prompt the participants. Although the focus groups were brief, they generated meaningful data. No minimum duration was set, and sessions concluded once all questions had been answered. To maintain pupil engagement, and to fit within school and study scheduling constraints, the discussions were intentionally kept concise. The focus groups were audio recorded and transcribed verbatim. These transcriptions were used for inductive semantic thematic analysis to identify key themes throughout the focus group. This approach was taken previously with a similar study population [16].

### 2.6. Data Analysis

Data were analyzed using Statistical Package for the Social Sciences (SPSS) (Version 30; SPSS Inc., Chicago, IL., USA). Paired Samples T-tests and Wilcoxon Signed Rank Tests (for non-parametric data) were conducted to analyze the differences in distance covered (m), physical activity enjoyment and average heart rate between The Daily Mile Normal and The Daily Mile Self-Control trial. These variables were collected once per participant in each trial, allowing for within-participant comparison.

Cognitive data (response time and accuracy) were analyzed using R (version 4.5.2; https://www.r-project.org). First, response times were filtered, in line with Cooper et al. [46], with minimum (100 ms) and maximum (2000–10,000 ms, depending on task complexity) cut-offs applied. This allowed for any unusually slow or fast responses to be excluded. Linear mixed effects models (with random intercepts per participant) were used to examine the interaction effects (trial × time interaction) on (a) the effects of physical activity (The Daily Mile Normal and The Daily Mile Self-Control) compared to a resting control, and (b) the effects of the addition of the self-control activities (The Daily Mile Self-Control) compared to The Daily Mile Normal. Additionally, the mixed-effects models allowed individual variability to be modelled appropriately. Response time analyses were performed with the nlme package, which implements mixed effect models and yields *t* statistics. Accuracy analyses were performed using the lme4 package, which also implements mixed effect models for data with a binomial outcome distribution (i.e., accuracy data) and yields *z* statistics.

Focus groups were recorded and transcribed verbatim by a member of the research team, producing 165 pages in total. The transcripts were analyzed through inductive semantic thematic analysis [47]. Codes were identified and systematically allocated, upon multiple reviews of the transcripts from two authors.

## 3. Results

### 3.1. Cognitive Function

Response time and accuracy data at baseline, immediately post-activity and 45 min post-activity, across the control, The Daily Mile Normal, and The Daily Mile Self-Control trials, for each cognitive function test, are displayed in Table 2.

#### 3.1.1. Effect of Physical Activity Compared to Rest

**Stroop Test**

**Figure 2:**
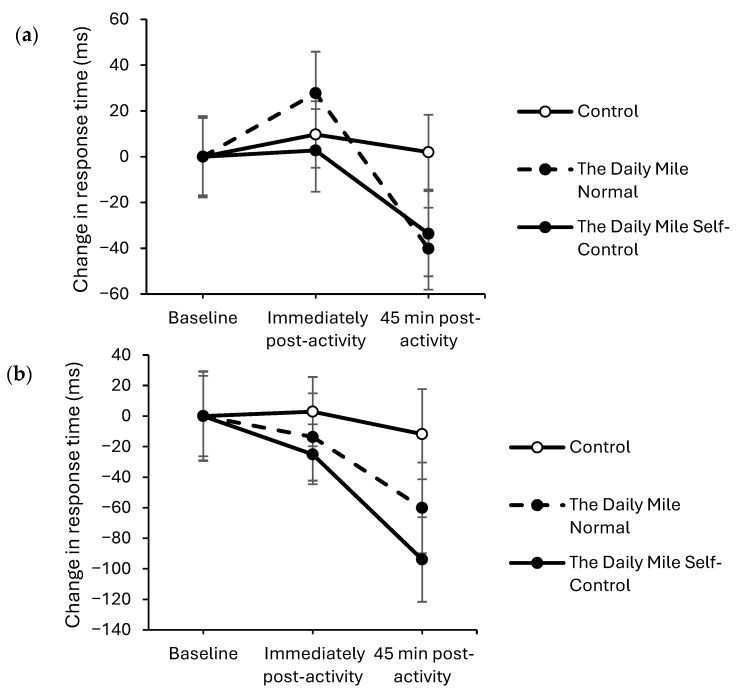
Change from baseline response times (ms) across the trial on the simple (trial × time, *p* < 0.001) (**a**) and complex (trial × time, *p* = 0.006) (**b**) levels of the Stroop test for physical activity (The Daily Mile Normal and The Daily Mile Self-Control) and Control (Resting) trials.

At 45 min post-activity, response times improved to a greater extent on the physical activity trials compared to the resting trial on both the simple (trial × time interaction, *t*_(16,331)_ = 3.77, *p* < 0.001; Figure 2a) and complex (trial × time interaction, *t*_(31,590)_ = 2.73, *p* = 0.006; Figure 2b) levels of the Stroop test. There was no effect of physical activity on response times immediately post-activity on either the simple (trial × time interaction, *p* = 0.855) or complex (*p* = 0.383) levels of the Stroop test.

**Figure 3:**
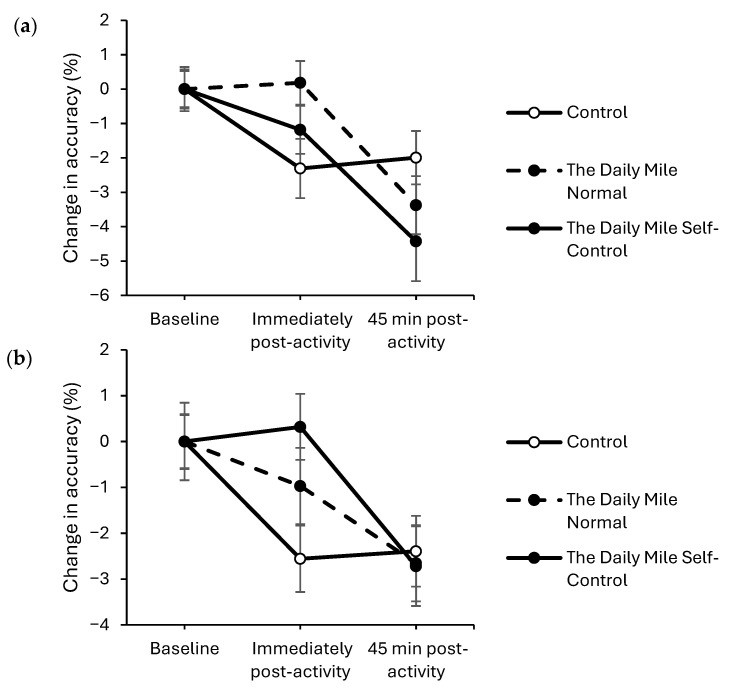
Change from baseline accuracy (%) across the trial on the simple (trial × time, *p* = 0.040) (**a**), and complex (trial × time, *p* < 0.001) (**b**) levels of the Stroop test, for physical activity (The Daily Mile Normal and The Daily Mile Self-Control) and Control (Resting) trials.

Accuracy was better maintained immediately post-activity on the physical activity trials compared to the resting trial, on both the simple (trial × time interaction, *z*_(17,775)_ = −2.059, *p* = 0.040, Figure 3a) and complex (*z*_(34,881)_ = −3.36, *p* < 0.001, Figure 3b) levels of the Stroop test. However, there was no effect of physical activity on accuracy 45 min post-activity on either level of the Stroop test (trial × time interactions, simple level: *p* = 0.072, complex level: *p* = 0.962).

**Sternberg**

**Figure 4:**
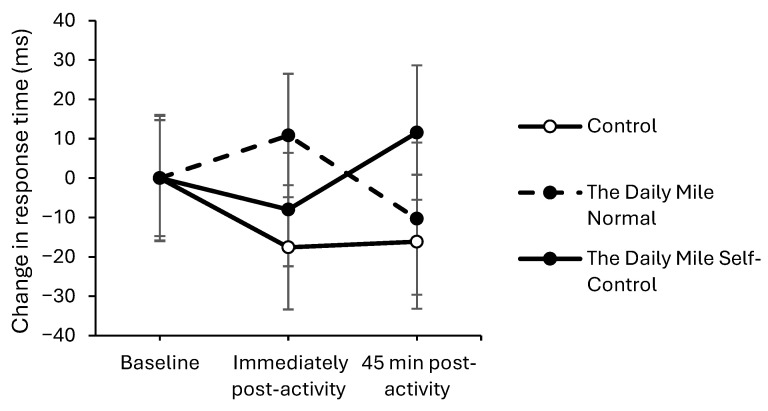
Change from baseline response times (ms) across the trial on the one-item level of the Sternberg Paradigm, for physical activity (The Daily Mile Normal and The Daily Mile Self-Control) and Control (Resting) trials (trial × time, *p* = 0.005).

On the one-item level of the Sternberg Paradigm, response times were maintained immediately post-activity on the physical activity trials; however the resting trial improved to a greater extent (trial × time interaction, *t*_(12,919)_ = −2.84, *p* = 0.005, Figure 4). There was no effect of physical activity on response times on the three-item or five-item levels of the Sternberg Paradigm either immediately post-activity (trial × time interactions: three-item, *p* = 0.121; five-item, *p* = 0.368) or all levels at 45 min post-activity (one-item: *p* = 0.09, three-item: *p* = 0.245, five-item: *p* = 0.314).

**Figure 5:**
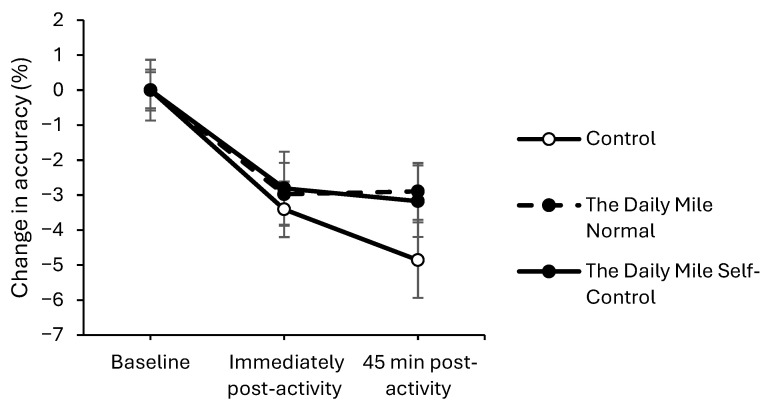
Change from baseline accuracy (%) across the trial on the three-item level of the Sternberg Paradigm, for physical activity (The Daily Mile Normal and The Daily Mile Self-Control) and Control (Resting) trials (trial × time, *p* = 0.012).

On the three-item level of the Sternberg Paradigm, accuracy was better maintained 45 min post-activity on the physical activity trials compared to the resting trial (trial × time interaction, *z*_(28,193)_ = −2.50, *p* = 0.012, Figure 5). However, there was no effect of physical activity on accuracy immediately post-activity, on any level of the Sternberg Paradigm (trial × time interactions, one-item level: *p* = 0.276, three-item level: *p* = 0.197, five-item level: *p* = 0.553). or 45 min post-activity on the one-item and five-item levels of the Sternberg Paradigm (one-item: *p* = 0.829, five-item: *p* = 0.213).

**Visual Search**

**Figure 6:**
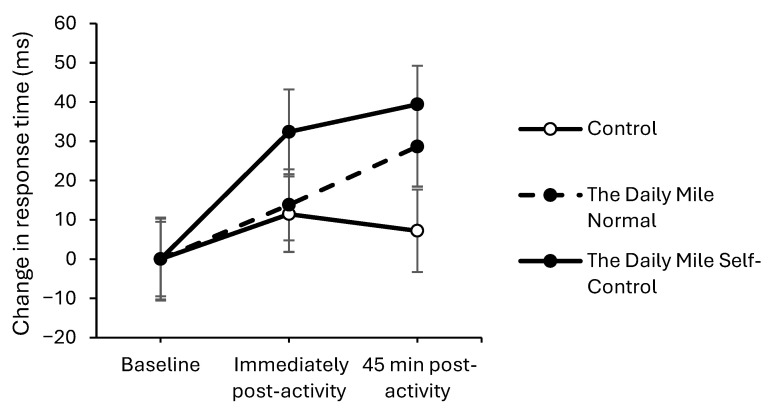
Change from baseline response times (ms) across the trial on the simple level of the Visual Search test, for physical activity (The Daily Mile Normal and The Daily Mile Self-Control) and Control (Resting) trials (trial × time, *p* = 0.022).

On the simple level of the Visual Search test, response times worsened to a greater extent on the physical activity trials immediately post-activity, compared to the resting trial (trial × time interaction, *t*_(18,376)_ = −2.285, *p* = 0.022, Figure 6). However, there was no effect of physical activity on response times immediately post-activity on the complex level (trial × time interaction, *p* = 0.695), or at 45 min post-activity on either level (simple level: *p* = 0.214, complex level: *p* = 0.636) of the Visual Search test. There were no significant trial × time interactions found for accuracy, on either the simple or complex level of the Visual Search test at immediately post-activity (simple: *p* = 0.104, complex: *p* = 0.662) or at 45 min post-activity (simple: *p* = 0.142, complex: *p* = 0.270).

#### 3.1.2. Effect of Self-Control

**Stroop Test**

There was no effect of the addition of self-control activities to The Daily Mile on response times immediately post-activity on either level (trial × time interactions, simple level: *p* = 0.182, complex level: *p* = 0.781) or at 45 min post-activity (simple level: *p* = 0.763, complex level: *p* = 0.265) of the Stroop test.

**Figure 7:**
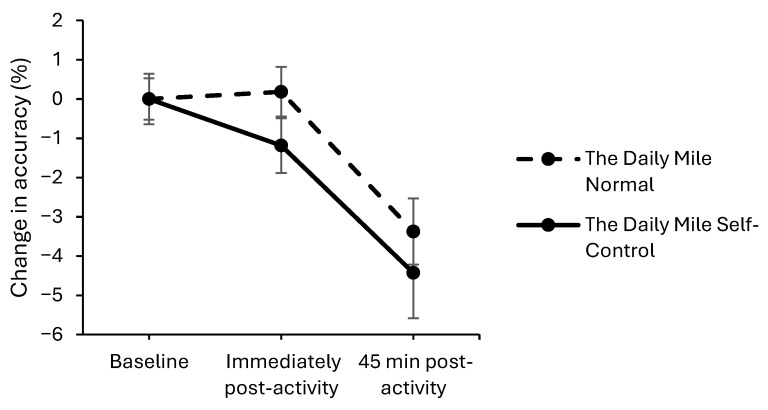
Change from baseline accuracy (%) across the trial on the simple level of the Stroop test, for The Daily Mile Normal and The Daily Mile Self-Control trials (trial × time, *p* = 0.045).

On the simple level of the Stroop test, accuracy declined to a greater extent on The Daily Mile Self-Control trial, compared to The Daily Mile Normal trial, immediately post-activity (trial × time interaction, (*z*_(11,801)_ = −2.00, *p* = 0.045, Figure 7). Additionally, at 45 min post-activity, accuracy declined to a greater extent on The Daily Mile Self-Control trial compared to The Daily Mile Normal trial on the simple level (*z*_(11,801)_ = −1.69, *p* = 0.092) of the Stroop test. There was no effect of the addition of self-control activities to The Daily Mile on accuracy immediately post-activity (*p* = 0.072) or 45 min post-activity on the complex level (*p* = 0.752).

**Sternberg**

**Figure 8:**
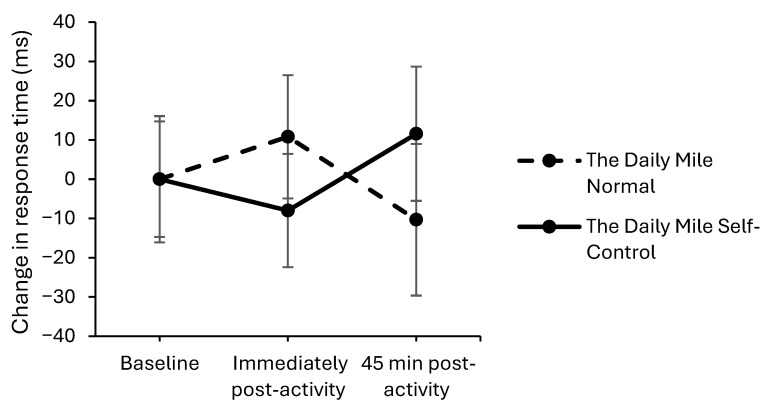
Change from baseline response times (ms) across the trial on the one-item level of the Sternberg Paradigm, for The Daily Mile Normal and The Daily Mile Self-Control trials (trial × time, *p* = 0.027).

On the one-item level of the Sternberg Paradigm, response times improved by a greater extent on The Daily Mile Self-Control trial compared to The Daily Mile Normal trial, immediately post-activity (trial × time interaction, *t*_(8521)_ = −2.22, *p* = 0.027, Figure 8). However, there was no effect of the addition of self-control activities to The Daily Mile on response times immediately post-activity on the three-item level (trial × time interaction, *p* = 0.205) and five-item level (*p* = 0.079), or at 45 min post-activity for all levels (one-item: *p* = 0.967, three-item: *p* = 0.527, five-item: *p* = 0.122) of the Sternberg Paradigm.

There was no effect of the addition of self-control activities to The Daily Mile on accuracy immediately post-activity (trial × time interaction, one-item: *p* = 0.429, three-item: *p* = 0.454, five-item: *p* = 0.056) or at 45 min post-activity, for all levels (one-item: *p* = 0.763, three-item: *p* = 0.972, five-item: *p* = 0.347) across all levels of the Sternberg Paradigm.

**Visual Search**

**Figure 9:**
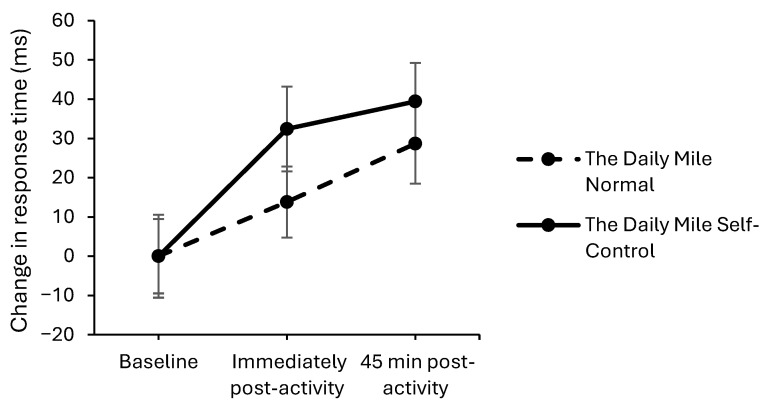
Change from baseline response times (ms) across the trial on the simple level of the Visual Search test, for The Daily Mile Normal and The Daily Mile Self-Control trials (trial × time, *p* = 0.016).

On the simple level of the Visual Search test, response times worsened to a greater extent on The Daily Mile Self-Control, compared to The Daily Mile Normal, both immediately (trial × time interaction, *t*_(12,204)_ = 2.41, *p* = 0.016, Figure 9) and 45 min (*t*_(12,204)_ = 2.76, *p* = 0.006) post-activity. No effect of the addition of self-control activities to The Daily Mile on response times was observed at immediately post-activity (*p* = 0.913) or 45 min post-activity (*p* = 0.079) on the complex level of the Visual Search test.

**Figure 10:**
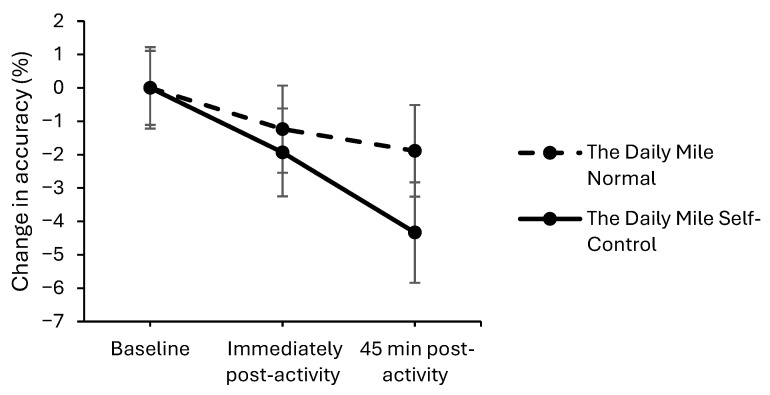
Change from baseline accuracy (%) across the trial on the simple level of the Visual Search test, for The Daily Mile Normal and The Daily Mile Self-Control trials (trial × time, *p* = 0.005).

On the simple level of the Visual Search test, accuracy declined to a greater extent on The Daily Mile Self-Control trial, compared to The Daily Mile Normal trial 45 min post-activity (trial × time interaction, *z*_(14,471)_ = −2.79, *p* = 0.005, Figure 10). No effect of self-control on accuracy was observed at immediately post-activity on the simple level (trial × time interaction, *p* = 0.365), or immediately post-activity (*p* = 0.091) or 45 min post-activity on the complex level of the Visual Search test (*p* = 0.868).

### 3.2. Physical Activity Enjoyment

There was no significant difference in physical activity enjoyment between The Daily Mile Self-Control trial and the Daily Mile Normal trial (*p* = 0.085).

### 3.3. Global Positioning Systems

There was no significant difference in distance covered during the Daily Mile activity between The Daily Mile Self-Control trial (2.10 ± 0.49 km) and The Daily Mile Normal trial (2.13 ± 0.41 km, *p* = 0.147).

### 3.4. Heart Rate

Average heart rate during the activity was significantly higher in The Daily Mile Self-Control trial (175 ± 16 beats min^−1^, 87 ± 8% HR_max_) than in The Daily Mile Normal trial (170 ± 15 beats min^−1^, 84 ± 8% HR_max_; *t*_(67)_ = −2.29, *p* = 0.025, *d* = −0.28, 95% CI [−0.52, −0.03]).

### 3.5. Focus Groups

To highlight participants perceptions and enjoyment towards The Daily Mile Normal and The Daily Mile Self-Control, the following results are organized around five key themes: (1) Motivation and engagement to participate in The Daily Mile Normal and The Daily Mile Self-Control; (2) Enjoyment and overall experience of The Daily Mile Normal and The Daily Mile Self-Control; (3) Social and autonomy factors shaping participation; (4) Activity design and variety within The Daily Mile Self-Control; (5) Optimizing The Daily Mile and self-control elements that were apparent from the focus group data, with multiple sub-themes within each (Table 3).

#### 3.5.1. Motivation and Engagement to Participate in the Daily Mile Normal and the Daily Mile Self-Control

This theme centers around the factors that influenced participation and incentivized enjoyment in The Daily Mile Normal and The Daily Mile Self-Control. Within this theme, six sub-themes were generated: (1) Drivers of engagement in The Daily Mile; (2) Sustaining momentum during The Daily Mile; (3) Motivation levels and willingness to exert effort; (4) Emotional responses to the physical activity; (5) Influence of competition on participation; (6) Cognitive engagement with the physical activity.

**Drivers of engagement in The Daily Mile**: Many participants noted that the games included in The Daily Mile Self-Control offered a more engaging approach to The Daily Mile, helping to prevent the monotony of The Daily Mile Normal: “*With the games was way more fun, but without the games it wasn’t really that interesting. But otherwise it* (The Daily Mile Normal) *just feels a bit too basic and a bit boring*” (Participant 210).

**Sustaining momentum during The Daily Mile**: Some participants highlighted how The Daily Mile Self-Control interrupted the running momentum and rhythm that they had developed across the session, as they had to engage in the games (e.g., play Simon Says), and instead would have preferred to have maintained a continuous pace for 15 min, like The Daily Mile Normal allows for:

“*I didn’t like doing the break because every time, because when I was going round I wasn’t stopping, so I wasn’t used to having a break, and then when I stopped running and started again, um, I felt like it was a bit harder*”

(Participant 201).

**Motivation levels and willingness to exert effort**: Many participants expressed how the inclusion of the games in The Daily Mile Self-Control was a key motivational factor to participate compared to The Daily Mile Normal, enhancing their willingness to engage and complete the session: “*I feel like I was a bit more motivated with games. Um, in a way, since it was kind of a bit more fun and appealing to me. So it kind of felt like I wanted to do that bit more*” (Participant 200).

**Emotional responses to physical activity**: Positive emotional responses were generated from participants regarding both The Daily Mile Normal and The Daily Mile Self-Control: “*For me it was also a bit of effort. I felt proud of myself*” (Participant 220). Showcasing that despite adapting The Daily Mile, it still positively influenced participants beliefs and attitudes towards the physical activity. However, some participants expressed negative feelings towards The Daily Mile Self-Control, due to a preference of not enjoying running and having less control of what to do during the games (i.e., having to run in the game run–walk):

“*I think I didn’t really like it that much because of when we need to walk and it makes I mean…but when you say walk we need to run. It just makes… it confuses me. So, it makes me have to run a lot and that’s what I don’t like about it*”

(Participant 156).

**Influence of competition on participation**: At a few schools, participants adopted a competitive approach, whereby they raced each other throughout the entirety of The Daily Mile Normal. This element of competition created a driver to engage in The Daily Mile Normal by a desire to avoid being overtaken in the competitive context: “*If there was no competition, I would have just sat out*” (Participant 118).

**Cognitive engagement to the physical activity**: Given the cognitively engaging element introduced into The Daily Mile Self-Control, in the form of self-control, researchers wanted to gain an understanding of the cognitive load that participants experienced between the two sessions. Several participants highlighted how the additional cognitive load in The Daily Mile Self-Control negatively impacted their enjoyment of the sessions, as the added focus required by the games was not well received: “*Because, with the games it’s like a bit confusing. Yeah, um, and like, I’m already running and focusing on, like, my breathing, and I don’t want on top of that the confusing games*” (Participant 205). However, other participants reported how they appreciated the challenge that came with additional cognitive engagement: “*I think it’s good because it confuses your brain*” (Participant 166).

#### 3.5.2. Enjoyment and Overall Experience of the Daily Mile Normal and the Daily Mile Self-Control

This theme captures the positive feelings and experiences associated with The Daily Mile Normal and The Daily Mile Self-Control, presented across three sub-themes: (1) General enjoyment associated with participation; (2) Perceived physical and mental benefits from participation; (3) Enjoyment driving perceived performance and confidence.

**General enjoyment associated with participation**: Overall, most of the participants responded positively to both The Daily Mile Normal and The Daily Mile Self-Control. In particular, a lot of participants expressed a fondness for The Daily Mile Self-Control, as it offered an alternative to standard running, which The Daily Mile Normal follows: “*I like doing it because like, when I, when I got tired, I would always have like something to do so it took my mind off running*” (Participant 199).

**Perceived physical and mental benefits from participation:** Participants reported feeling healthier and more active after taking part in both Daily Mile sessions. In particular, participants reported that The Daily Mile Self-Control left them feeling as though their muscles had been thoroughly worked, due to the inclusion of the self-control games: “*The one with the games, because we needed to touch our toes and … so we were doing different exercises, for like our different muscles*” (Participant 174). Additionally, some participants reported that subsequent concentration was enhanced following engagement in The Daily Mile Self-Control: “*It made me feel very active and energetic and made me feel concentrated on what I’m doing*” (Participant 191).

**Enjoyment driving perceived performance and confidence:** When asked what prompted the enjoyment in The Daily Mile Normal and The Daily Mile Self-Control, several participants indicated that, despite lacking a strong inclination to running, the sessions enabled them to engage in a novel activity, fostering increased confidence to step beyond familiar norms: “*I’m not really a runner though, but like it kind of put me out my comfort zone*” (Participant 107). This was also coupled with participants highlighting that the inclusion of games within The Daily Mile Self-Control altered their perception of their own performance:

“*Like it makes me feel like I’m running faster, even though I’m probably running a bit slower. It’s like it’s because, um, the games are in it, and it feels like I’m running at a normal pace. And because the games are in it, but I’ve actually slowed down a bit. So I feel like I’m going faster*”

(Participant 165).

This demonstrates how the enjoyment can mask physical exertion and enhance perceived capabilities. On the other hand, participants who favored The Daily Mile Normal perceived their performance as superior in The Daily Mile Normal sessions compared to The Daily Mile Self-Control: “*I prefer the normal one because I felt the game one was like distracting me from like doing that running. I felt like I did the better on the normal one than the game one*” (Participant 197). Suggesting that enjoyment influenced perceived performance, with participants feeling they performed better in sessions they preferred, irrespective of the inclusion of games.

#### 3.5.3. Social and Autonomy Factors Shaping Participation

This theme refers to interpersonal and self-directed influences that help shape participants’ willingness, confidence and overall engagement in The Daily Mile. Two sub-themes were developed: (1) Role of social interaction and peer influence; (2) Preference for autonomy.

**Role of social interaction and peer influence**: In line with one of the ten core principles of The Daily Mile of promoting social interaction, participants highlighted how they were able to exercise with their friends. In particular, a greater number of participants reported that The Daily Mile Normal facilitated social interaction with peers more effectively than The Daily Mile Self-Control, as the absence of game-related interruptions allowed continuous engagement and physical activity with friends: “*I like the one without the games, because I could just run with my friends without focusing on the games*” (Participant 203).

**Preference for autonomy:** Participants expressed contrasting views regarding autonomy within The Daily Mile sessions. Some valued the opportunity to self-regulate their pace and concentrate on their individual performance, making The Daily Mile Normal more appealing: “*Err, I preferred to do it without the game because I felt like… I’d rather just focus on my running… rather than when I’m really tired, focusing on doing other things*” (Participant 201). Conversely, others preferred the structured nature that The Daily Mile Self-Control offers, particularly the games that provided explicit instructions, as this reduced reliance on self-motivation and offered a more guided experience: “*I was probably more motivated to run during the games because you would tell us to run, so we had to do it*” (Participant 182).

#### 3.5.4. Activity Design and Variety Within the Daily Mile Self-Control

The following theme addresses The Daily Mile Self-Control, focusing specifically on the games included. Four sub-themes were generated: (1) Preference for incorporating game-based activities; (2) Importance of variety; (3) Age appropriateness and suitability of self-control games; (4) Advantages and challenges with game difficulty.

**Preference for incorporating game-based activities**: Whilst most participants reported positive views towards both Daily Mile sessions, many did prefer The Daily Mile Self-Control, as it served as a distraction from the fatigue of continuous running and introduced an added challenge, which they found to be enjoyable: “*You weren’t focused on how tired you were when you run around, you were just more focused on playing the games*” (Participant 101).

**Importance of variety:** When asked about what the participants thought of The Daily Mile Self-Control games specifically, several reported that whilst the games offered something new and enjoyable, if they were to continue with them in the future, they would like to have more games to choose from, to prevent the initiative becoming monotonous and repetitive. Participants suggested that a resource bank of games to choose games from would keep The Daily Mile exciting and engaging:

“*Yeah, because honestly, if you would do something five times a day other… same, like basically the same thing, it would get boring. But if you change it once in a while, it’s like a new experience, so it will be fun*”

(Participant 220).

**Age appropriateness and perceived suitability of self-control games:** The games were designed and selected for primary school age children. Many participants expressed that they enjoyed the games and considered them appropriate for their age group. However, certain games were favored more in general than others. Simon Says overall was considered the most appropriate game for all the participants, whereas body-part-mix-up was less so: “*It’s a good game for older kids and younger kids” Referring to Simon Says* (Participant 116).

**Advantages and challenges with difficulty:** Participants were asked how challenging they found The Daily Mile Normal and The Daily Mile Self-Control, and despite the difficulty of The Daily Mile Self-Control being higher, some participants highlighted that as a preferable thing: “*I liked how it wasn’t just running. They made the games like tricky*” (Participant 155). On the other hand, there were some participants who did not appreciate the complexity of the games and instead would have just preferred to engage in The Daily Mile Normal: “*Regular, because you can’t get confused*” (Participant 154).

#### 3.5.5. Optimizing the Daily Mile and Self-Control Elements

This theme focuses on elements of The Daily Mile Normal and The Daily Mile Self-Control that participants felt could be improved to further its potential. Within this theme, three sub-themes were developed: (1) How The Daily Mile could be improved or adapted; (2) Suggestions for enhancing the self-control games; (3) Preferred duration of the self-control games.

**Ways The Daily Mile could be improved or adapted:** When asked what participants thought could be improved with either of The Daily Mile sessions, many participants wanted to change the physical activity from running, instead opting for alternative activities or sports (e.g., dribbling with a ball, highlighting a preference for something aside from running): “*We could have done like different like kind of sport, not running like you could do…dribbling*” (Participant 134). In addition, some participants reported how the structure of The Daily Mile Self-Control session could be altered, to enhance performance on The Daily Mile: “*I think I’d rather do the games beforehand because after the running, I’ll be more tired*” (Participant 201).

**Suggestions for enhancing the self-control games:** When asked how the self-control games could be improved, participants suggested introducing even more variety to avoid repetition and prevent boredom, such as rotating different games each week: “*I would like it to be like mixed up so every week we do it, you get different games*” (Participant 195). Additionally, a few participants recommended incorporating more elements into the games that increase complexity to make the activities more challenging.

**Preferred duration of the self-control games**: Participants expressed varied opinions regarding the preferable duration of the self-control games. Those who enjoyed the games preferred allocating more time to these activities and reducing the proportion of self-paced running within The Daily Mile: “*I actually feel like you should add a bit more time, because it’s quite fun*” (Participant 210). In contrast, participants who were less enthusiastic about the self-control component advocated for shorter game durations or their complete removal. Additionally, some participants suggested that approximately 30 s per game was ideal, as this maintained alignment with the self-paced nature of the session while introducing a brief, engaging game.

Overall, participant’s perceptions of The Daily Mile Normal and The Daily Mile Self-Control were varied. Those with preference for The Daily Mile Normal highlighted feelings of independent control to pace themselves and to not overcomplicate the initiative through the inclusion of games with rules in. On the other hand, those with preference for The Daily Mile Self-Control expressed feelings of enjoyment to having a diverse and varied experience during the session, by including games. Additionally, the cognitively challenging nature of the games was responded to mostly positively.

## 4. Discussion

The main findings of the present study indicate that when compared to rest, an acute bout of The Daily Mile improved inhibitory control and helped maintain accuracy in working memory, but perception showed a temporary decline immediately post-activity. Furthermore, when self-control tasks were added to The Daily Mile, working memory response times improved, but accuracy in inhibitory control and perception declined, particularly immediately post-activity. When asking children for their perceptions of the interventions, they reported physical and mental benefits, enhanced confidence, and social interaction as facilitators of enjoyment. Notably, many participants preferred The Daily Mile when self-control activities were incorporated, reporting greater enjoyment, variety, challenge, and distraction from fatigue.

Across cognitive domains, the present study showed that an acute bout of physical activity (The Daily Mile) produced positive but domain specific, effects on cognition. Specifically, inhibitory control was the cognitive domain demonstrating the greatest benefit, with improved response times (45 min post-activity) and better maintained accuracy. This finding is consistent with previous evidence [14,48], which identified inhibitory control and executive function as the domains showing the most consistent beneficial effects of physical activity. Furthermore, accuracy in inhibitory control was better maintained in the physical activity trials compared to rest. This aligns with Hatch et al. [16], who similarly found a tendency for improved accuracy following The Daily Mile, suggesting that physical activity may help preserve inhibitory control and reduce cognitive fatigue. Working memory demonstrated a similar pattern of maintained accuracy following the physical activity trials compared to rest. Specifically, on the three-item level of the Sternberg Paradigm and 45 min post-activity consistent with findings from previous work [14,16], Paschen et al. [49] reported improvements in working memory speed, but not accuracy, for low cognitive demanding physical activity versus rest. Such differences between the present study and Paschen et al. [49] reflect a variation in the cognitive demands of the working memory tasks used across studies. In the present study, physical activity appeared to help maintain working memory accuracy relative to rest, suggesting methodological differences rather than contradictory effects of activity account for the mixed findings. In contrast, perceptual performance declined to a greater extent immediately post-activity compared to the rest, aligning with Connell et al. [50], who reported that moderate daily exercise performed immediately prior to a visual perceptual task (motion direction discrimination task) may have impaired learning. Both their study and the present study assessed perception; however, Connell et al. [50] used tasks that tap into different components of visual processing. Therefore, the present study extends these findings by suggesting that perceptual performance may be inhibited following activity. Together, the findings of the current study indicate that an acute bout of physical activity does not influence all cognitive domains uniformly. Instead, its benefits appear for higher-level executive functions, in which perceptual performance may be more sensitive to immediate post-activity fatigue.

To the authors’ knowledge, this is the first study to examine the effect of additional self-control activities in The Daily Mile on cognition. Adding self-control tasks into The Daily Mile appeared to impair inhibitory control accuracy compared to The Daily Mile Normal. This suggests inclusion of self-control tasks may hinder short-term inhibitory control. This aligns with the Strength Model of Self-Control [23], which proposes that self-control is a limited resource. Following The Daily Mile Self-Control, participants self-control reserves may have become sufficiently depleted so that they were unable to exert further self-control on the cognitive tasks; thus performance decreased. Working memory displayed mixed responses following The Daily Mile Self-Control. Low-demand working memory tasks (one-item Sternberg) saw greater response time improvements following The Daily Mile Self-Control, compared to The Daily Mile Normal. Whereas the high-demand tasks (five-item Sternberg) saw a reduction in response times, suggesting potential benefits for speed under certain demand conditions. Working memory accuracy did not differ between The Daily Mile Normal and The Daily Mile Self-Control, contrasting with Biino, Tinagli, Borioni, and Pesce [51], whose younger participant sample may have greater developmental plasticity [52]. Furthermore, perceptual performance declined following The Daily Mile Self-Control compared to The Daily Mile Normal, consistent with evidence that suggests that cognitive exertion can temporarily impair perceptual processing [53]. Overall, the present study provides initial evidence that including self-control activities into physical activity interventions may be beneficial for some aspects of cognition. However, this should be done with an awareness that such activities may deplete self-control and lead to temporary declines in cognition, acutely post-activity. The longer-term (chronic) effects of such activity warrant further investigation.

The Daily Mile evoked positive emotions and boosted confidence for some participants. This is of particular importance as higher exercise self-efficacy has been linked to stronger intentions to be physically active [54]. The present study extends previous knowledge by highlighting the factors that influence children’s engagement in The Daily Mile. Specifically, social interaction and perceived physical and mental health benefits emerged as key motivators, consistent with Hatch et al. [16]. Sustaining these positive experiences is therefore crucial for maintaining engagement with The Daily Mile. Many participants appreciated the variety introduced through the inclusion of the self-control tasks and expressed a preference for more activities. This finding builds on one of the challenges reported by Hatch et al. [16], of making the activity less monotonous and boring. The self-control tasks appeared to reduce boredom by providing a distraction and shifting focus away from physical effort. Similar findings were reported by Hanna et al. [55], where incorporating games within The Daily Mile helped sustain engagement and enjoyment, further supporting the value of variety. The cognitive demands of The Daily Mile Self-Control resulted in positive responses in the form of heightened enjoyment, with participants appreciating the added challenge. However, some participants noted contrasting perceptions, whereby they felt that the self-control tasks disrupted their momentum, potentially blunting their physical activity enjoyment. This contrasts with previous literature suggesting that continuous exercise results in lower enjoyment compared to interval-based physical activity [56]. It emphasizes how modality, intensity, and duration preferences differ between individuals; demonstrating a one-size-fits-all approach is not always appropriate. Participants also reported a lack of autonomy in The Daily Mile Self-Control. As Hatch et al. [16] identified autonomy in The Daily Mile as a key factor in its success, eliminating this element likely contributed to the negative responses. It is also worth noting that some participants preferred clear instructions and not needing to make decisions, again emphasizing the importance of accounting for individual differences.

Another key finding of the present study was that perceived enjoyment was different between The Daily Mile Normal and The Daily Mile Self-Control. This aligns with the mixed focus group responses discussed above and may reflect the study’s within-subjects design, where a single exposure to each condition may not have been sufficient to generate noticeable differences. Contrastingly, Hanna et al. [55] reported higher physical activity enjoyment in an adapted Daily Mile group (using games such as relays and tag within The Daily Mile) compared to normal, likely due to their 10-week, between-subjects design, which may have reduced short-term variability. It could be suggested that long-term implementation of The Daily Mile Self-Control could lead to less boredom, and thus greater enjoyment and adherence to the activity.

As previously mentioned, the reduction in inhibitory control accuracy following engagement in The Daily Mile Self-Control may reflect a temporary depletion of regulatory resources. This interpretation aligns with the qualitative findings, in which many participants favored The Daily Mile Normal due to its simplicity and the reduced mental effort required. If The Daily Mile Self-Control taxes these resources, this may explain both the reduced post-activity accuracy and the preference for the less cognitively demanding Daily Mile. Furthermore, the decline in perceptual accuracy is consistent with participants’ perceptions on the self-control tasks requiring more mental effort and disrupting their momentum, suggesting that additional cognitive demands could leave fewer resources available for perceptual processing. This highlights how autonomy and perceived mental effort can shape performance on both inhibitory-control and perceptual tasks. Alternatively, despite The Daily Mile Self-Control imposing greater cognitive demands, low-demand working memory was maintained with response times improving. For those participants who enjoyed The Daily Mile Self-Control, citing reduced boredom and an added challenge as to reasons why, the self-control tasks may have increased arousal and attentional focus, facilitating improved working memory performance.

The present study introduced a novel modification of adding self-control activities to The Daily Mile, with the aim to increase participant enjoyment whilst maximizing cognitive benefits. The mixed methods design provided a richer interpretation of results, an understanding of participant experience, and highlighted areas to further enhance The Daily Mile intervention. However, the present study is not without limitation. Only the acute cognitive effects were examined, and the acute effects reported may not translate into chronic effects. Furthermore, the mixed cognitive results could be explained by the acute activity, whereby the effects on cognition can be highly variable and sensitive to individual differences. Therefore, future research should examine the longer-term effects of implementation on cognition, alongside how longer-term participation influences perceptions and enjoyment to inform the feasibility and sustainability of the intervention. The variability in the cognitive outcomes could be due to not externally controlling for exercise intensity, as explained by the significant difference in heart rate between the Daily Mile trials. However, the physical activity was intentionally designed to reflect the real-world application of The Daily Mile; thus the self-paced intensity was retained for ecological validity. Additionally, the repeated exposure to the cognitive tasks may have introduced learning effects, through improvements due to practice and task recognition. However, the study’s order-balanced design would have helped to mitigate this effect. A further limitation is that the self-control demands of The Daily Mile self-control tasks were not directly measured. Although the self-control activities were designed to include elements involved with response inhibition, we cannot quantify the precise level of cognitive demand imposed. As a result, interpretations regarding the cognitive complexity of the activities should be made with caution.

The findings of the present study help to offer a basis for future research. Qualitative findings indicated varied participant experiences in The Daily Mile sessions, which could be explained by variations in fitness levels. Low-fit participants may have exerted more effort, limiting their ability to reap the cognitive benefits compared with higher-fit peers. This aligns with Hatch et al. [16], who reported those with higher cardiorespiratory fitness demonstrated faster response times in cognitive tasks. These observations further support the notion that adapting physical activity to individual needs and preferences may enhance cognitive benefits; thus, future research should look to tailor physical activity to suit individual fitness levels.

Based on the findings of the current study, practical implications can be drawn. Specifically, schools could integrate self-control-focused versions of The Daily Mile into the school timetable as a low-cost, accessible way to support pupils’ cognitive functioning and provide a variety of activities to maintain engagement and enjoyment. Furthermore, offering both The Daily Mile Normal and The Daily Mile Self-Control to school children could accommodate all differences in fitness, enjoyment and activity preferences, whilst still enhancing cognitive function.

## 5. Conclusions

This study is the first to examine the effect of incorporating self-control tasks into The Daily Mile on cognition in children. The findings suggest that physical activity may benefit higher-order cognition, relative to rest, while the addition of self-control tasks into The Daily Mile elicits mixed cognitive and qualitative responses, compared to The Daily Mile Normal. Tailoring physical activity to individual preferences may therefore optimize engagement and cognitive benefits. Future research should explore the long-term effects of The Daily Mile Self-Control, to determine how long-term participation affects cognition and shapes enjoyment to help inform intervention implementation.

## Figures and Tables

**Table 1:** Participant anthropometric characteristics (Mean ± SD).

	Overall (n = 99)	Boys (n = 49)	Girls (n = 50)
Age (y)	10.2 ± 1.1	10.4 ± 1.1	10.1 ± 1.1
Height (cm)	143.2 ± 9.9	144.7 ± 8.9	141.7 ± 10.7
Body mass (kg)	37.4 ± 9.7	38.0 ± 10.5	36.9 ± 9.0
Body mass index (BMI; kg.m^2^)	18.0 ± 3.2	18.2 ± 3.5	18.0 ± 3.0
BMI Percentile ^a^	56.84 ± 33.35	56.00 ± 33.03	57.67 ± 34.00
Maturity offset (y) ^b^	−2.08 ± 1.06	−2.56 ± 0.80	−1.61 ± 1.07
Waist circumference (cm)	63.3 ± 8.8	63.3 ± 10.0	63.3 ± 7.6
Hip circumference (cm)	76.9 ± 8.3	76.9 ± 9.0	76.8 ± 7.7
MSFT Distance (m)	700 ± 380	840 ± 420	560 ± 260

^a^ Calculated based on national reference values [34]. ^b^ Calculated using the method by Moore et al. [35].

**Table 2:** Cognitive function data across the activity (Daily Mile and Daily Mile Self-Control) and control trials. Data are Mean ± S.E.M.

Test	Level	Variable	Control Trial			The Daily Mile Normal Trial			The Daily Mile Self-Control Trial		
			Baseline	Immediately post-activity	45 min post-activity	Baseline	Immediately post-activity	45 min post-activity	Baseline	Immediately post-activity	45 min post-activity
Stroop test	Simple	Response time (ms)	876 ± 17	885 ± 20	873 ± 18	905 ± 18	933 ± 17	863 ± 19	907 ± 17	908 ± 20	859 ± 21
		Accuracy (%)	96.8 ± 0.6	94.5 ± 0.8	94.8 ± 0.8	96.4 ± 0.6	96.6 ± 0.6	93.1 ± 1.0	97.4 ± 0.5	96.2 ± 0.6	93.0 ± 1.3
	Complex	Response time (ms)	1204 ± 29	1204 ± 33	1192 ± 35	1277 ± 29	1252 ± 29	1180 ± 29	1242 ± 26	1233 ± 30	1186 ± 35
		Accuracy (%)	95.2 ± 0.6	92.7 ±0.9	92.8 ± 0.9	95.0 ± 0.6	94.0 ± 1.0	92.4 ± 1.0	93.9 ± 0.8	94.4 ± 0.9	91.3 ± 1.2
Sternberg Paradigm	One-item	Response time (ms)	648 ± 16	631 ± 17	632 ± 17	652 ± 15	660 ± 16	642 ± 17	653 ± 16	645 ± 17	665 ± 20
		Accuracy (%)	95.5 ± 0.8	93.7 ± 0.9	93.1 ± 1.2	95.4 ± 0.6	95.3 ± 0.7	93.0 ± 1.1	95.5 ± 0.7	94.6 ± 0.9	93.3 ± 1.1
	Three-item	Response time (ms)	874 ± 24	847 ± 24	847 ± 23	897 ± 26	862 ± 21	875 ± 20	859 ± 21	873 ± 23	876 ± 26
		Accuracy (%)	95.4 ± 0.5	92.2 ± 0.8	90.7 ± 1.2	95.0 ± 0.6	92.2 ± 1.0	92.2 ± 0.8	94.4 ± 0.9	91.5 ± 1.1	91.4 ± 1.2
	Five-item	Response time (ms)	993 ± 21	969 ± 24	981 ± 30	988 ± 19	971 ± 20	956 ± 23	1017 ± 24	996 ± 22	957 ± 26
		Accuracy (%)	89.1 ± 1.1	86.2 ± 1.4	82.9 ± 1.6	89.4 ± 1.1	85.8 ± 1.4	84.2 ± 1.4	87.7 ± 1.4	86.5 ± 1.3	82.7 ± 1.7
Visual Search test	Simple	Response time (ms)	654 ± 10	666 ± 12	662 ± 10	657 ± 10	671 ± 11	684 ± 11	652 ± 11	684 ± 12	690 ± 12
		Accuracy (%)	91.9 ± 1.0	87.8 ± 1.2	87.0 ± 1.3	89.9 ± 1.2	88.6 ± 1.4	88.3 ± 1.3	90.8 ± 1.1	88.8 ± 1.4	86.5 ± 1.6
	Complex	Response time (ms)	1639 ± 48	1670 ± 51	1637 ± 51	1689 ± 53	1672 ± 54	1728 ± 54	1776 ± 54	1719 ± 56	1721 ± 60
		Accuracy (%)	88.6 ± 1.5	86.8 ± 1.7	85.4 ± 1.7	86.8 ± 1.7	84.3 ± 2.0	84.9 ± 1.8	86.7 ± 1.5	85.7 ± 1.9	83.5 ± 1.9

**Table 3:** Themes and sub-themes referring to elements influencing participants’ perceptions and enjoyment of The Daily Mile Normal and The Daily Mile Self-Control.

Theme	Sub-Theme	Quotes
Motivation and engagement to participate in The Daily Mile	Drivers of engagement in The Daily Mile	“instead of just running around, it was like planned running, so it actually felt like it had a purpose.” Participant 220
	Sustaining momentum during The Daily Mile	“I didn’t really like the break … kind of lost my rhythm and like if I was tired, then I could choose when I wanted to walk, in that I could… I just had to walk. So, like if I started to be able to like, really get a good rhythm and then I had to walk it” Participant 193
	Motivation levels and willingness to exert effort	“Weren’t as tiring as when we didn’t do the games. It motivated you a bit more” Participant 135
	Emotional responses to physical activity	“Running without the games was very boring. Because like, there’s nothing fun to do in it, it’s just running” Participant 216
	Influence of competition on participation	“Yeah, that’s why, that’s why races are so good. Because like you can get like, like you could do it like you could do. You have got them. So, like a group could go there. Then they would stay moving like, by like jogging like round waiting for their go.” Referring to a race element the students added in on The Daily Mile Normal Participant 113
	Cognitive engagement to the physical activity	“I feel like I worked harder in the games because I had to concentrate and run at the same time and I can talk and run, but I just can’t concentrate at and run at the same time, so it’s hard to concentrate” Participant 179
Enjoyment and overall experience of The Daily Mile Normal and The Daily Mile Self-Control	General enjoyment associated with participation	“I think I feel better without the games, but I enjoy it more with the games” Participant 169
	Perceived physical and mental benefits from participation	“It made me feel healthier. Because I feel like I do too much time sitting down on my phone” Participant 216 “That was also good for our communicating” [Referring to run-walk] Participant 135
	Enjoyment driving perceived performance and confidence:	“Kind of like good, because like everyone did, it kind of thing. Like everyone tried the best, no matter like if you were like, if you weren’t really confident in, like, running further, everyone tried.” Participant 109
Social and autonomy factors shaping participation	Role of social interaction and peer influence	“It did kind of help me because you didn’t want to get lapped, so I just sprinted like 2 laps.” Participant 123
	Preference for autonomy	“So I just think we could like do, do whatever we want, like pace ourselves. Do it like… we’re not told to be stopping and going” Participant 188
Activity design and variety within The Daily Mile Self-Control	Preference for incorporating game-based activities	“I preferred the one with the games… it’s because running itself can be quite boring. And then when you put the games in it, it makes it fun” Participant 176
	Importance of variety	“It was just running that we do that every day almost and we got to do games which made it more exciting and fun and more enjoyable” Participant 187
	Age appropriateness and perceived suitability of self-control games	“Was a bit boring… I would prefer just like running…it’s quite annoying…playing our head and toes game” Participant 136
	Advantages and challenges with difficulty	“I think I’d rather just run normally. Not overcomplicate things” Participant 201
Optimizing The Daily Mile and self-control elements	Ways The Daily Mile could be improved or adapted	“I think it would be good because some people like the running. Some people like the games. So you could choose if you wanted to do the games and choose if you didn’t want to do the games. Participant 193
	Suggestions for enhancing the self-control games	“If it was more other body parts, I probably would have liked it” Participant 164
	Preferred duration of the self-control games	“Maybe a bit too long because I didn’t have enough time to properly run” Participant 112

## Data Availability

Supporting data are available from the corresponding author upon reasonable request. Our dataset contains information about minors, including (but not limited to) age, gender, and health-related details. Many of the variables in the data set are considered sensitive personal data. Although the data has been pseudonymised, full public release could still pose a risk of re-identification, therefore data will be available upon reasonable request.
